# Dynamic Balance, but Not Precision Throw, Is Positively Associated with Academic Performance in Children †

**DOI:** 10.3390/ijerph17082790

**Published:** 2020-04-17

**Authors:** Rodrigo A. Lima, David F. Stodden, Karin A. Pfeiffer, Lisbeth R. Larsen, Mauro V. G. Barros, Anna Bugge, Lars B. Andersen

**Affiliations:** 1 Institute of Sport Science, University of Graz, Mozartgasse 14, 8010 Graz, Austria; 2 CAPES Foundation, Ministry of Education of Brazil, Brasília-DF 70040-020, Brazil; 3 Department of Physical Education & Athletic Training, University of South Carolina, Wheat Street, Suite 218, Columbia, SC 29208, USA; stodden@mailbox.sc.edu; 4 Department of Kinesiology, Michigan State University, East Lansing, MI 48824, USA; kap@msu.edu; 5 Social Education, Svendborg, Faculty of Education and social sciences, UCL University College, Niels Bohrs Allé 1, 5230 Odense M, Denmark; lrla@ucl.dk; 6 School of Physical Education, University of Pernambuco, Campus Universitario HUOC-ESEF, Arnobio Marques 310, Santo Amaro, Recife PE 50.100-130, Brazil; mauro.barros@upe.br; 7 Department of Midwifery, Physiotherapy, Occupational Therapy and Psychomotor Therapy, Faculty of Health Sciences, University College Copenhagen, 2200 Copenhagen N, Denmark; abug@kp.dk; 8 Faculty of Education, Arts and Sport; Western Norway University of Applied Sciences, Campus Sogndal, 6861 Sogndal, Norway; lars.bo.andersen@hvl.no

**Keywords:** academic success, adiposity, adolescents, mediation analysis, psychomotor performance

## Abstract

We analyzed the longitudinal association between dynamic balance and throwing skill with academic performance and whether waist circumference mediated these relationships. The current one-year longitudinal study followed 1020 first (mean age 7.87 ± 0.34 years) through fifth grade (mean age 11.87 ± 0.37 years) children, measured twice in 2010 and 2011. Dynamic balance and precision throw were measures of motor competence. Waist circumference was measured with a measuring tape at the umbilicus level. Academic performance was assessed by a combined score of standardized Danish language and math tests. Structural equation modeling was used for analysis. All coefficients are standardized. Balance was associated with academic performance when both sexes were combined (β = 0.126, 95% CI: 0.074 to 0.179), and waist circumference partially mediated the relationship (β = 0.021, 95% CI: 0.008 to 0.034). For boys, balance was associated with academic performance, but waist circumference did not mediate the association. For girls, balance presented direct, mediated (via waist circumference), and total associations with academic performance. Dynamic balance is an important gross motor function that was longitudinally related to academic performance, and waist circumference partially mediated the relationship. Precision throw was not found to be associated with academic performance in both sexes combined or when analyzed separately.

## 1. Introduction

The ability to move is essential for engaging in physical activities, especially in early childhood [1]. Acquiring and refining the ability to move involves complex interactions within the environment and the neuromuscular systems, and it is often defined as motor competence [2]. Typically, motor competence can be subdivided into gross and fine motor skills. Gross motor skills are related to movement and coordination of large body parts and movements such as the arms, legs, and trunk. On the other hand, fine motor skills are involved in smaller movements in the wrists, hands, fingers and feet [2].

The parallel development in gross motor performance and various aspects of brain function has been investigated in different lines of research, and evidence of the relationship between motor competence and cognitive function and academic performance has been demonstrated. A review from 2013 summarized the literature in this area and concluded that higher levels of fine motor competence, measured by the “Movement Assessment Battery for Children-MABC” battery, were associated with shorter reaction time in the Stroop task [3], which is a measure of selective attention and cognitive flexibility but not associated with the Go/NoGo test measuring response inhibition and sustained attention in children and adolescents [3]. One study in preschool children found that agility was positively associated with item memory cross-sectionally, and balance was associated with item memory longitudinally [4]. Better gross motor competence has been longitudinally associated with working memory in a group of 6–11-year-old children [5], and process-oriented movement has been positively associated with item memory in 5–6-year-old children [6]. In summary, evidence suggests that fine and gross motor competences are associated with inhibition and memory in children and adolescents [3].

The motor competence-academic performance relationship has also been investigated in cross-sectional, longitudinal and experimental investigations [7,8,9,10,11,12,13]. Overall, cross-sectional studies have reported equivocal results on the relationship between motor competence and academic performance. Lopes et al. (2013) and Esteban-Cornejo et al. (2014) observed a positive association between gross motor competence and academic performance, assessing gross motor competence by the KTK battery [7] and the 4 × 10 m shuttle run test [13], respectively. In contrast, Fernandes et al. (2016) observed that the 5 × 5 m shuttle run test was not associated with academic performance, yet hand-eye coordination was [8]. The contradictory results of cross-sectional research warrant further investigation of associations over time. 

Two longitudinal studies have reported inconsistent findings on the relationship between motor competence and academic performance. Haapala et al. (2014) followed children from seven to nine years of age and reported that speed and agility (assessed by the 5 × 5 m shuttle run test) and manual dexterity (measured by the box and block test) were longitudinally associated with academic performance in children, but static balance was not [9]. In addition, most of the associations were consistently observed in boys, but associations in girls were weaker or non-significant [9]. In contrast, Grissmer et al. (2010) [10] followed kindergarten children for three years and observed negative longitudinal association between gross motor competence (measured by skipping, hopping on one foot, walking backwards, and standing on one foot) and reading scores. In contrast, fine motor competence (measured by building blocks and drawing a person) was positively associated with both reading and math scores. Therefore, observational studies provide inconsistent evidence of a selective association between academic performance and specific motor competence constructs, gender, or age during childhood [9,10].

Two different investigations from the same school-based intervention observed positive effects of motor competence training during school hours on academic performance [11,12]. The first study evaluated the three-year effects of extra physical education lessons, 225 min/week for the intervention group compared to children exposed to the standard 90 min/week. During the intervention, children were evaluated by the “Motor skills Development as Basis for Learning (MUGI)” observation program [11], and, if necessary, children in the intervention group were exposed to an extra 60 min/week of motor competence training [11]. Both intervention activities focused on stimulating the development of motor competence. A lower rate of children in the intervention group presented motor competence deficits after the intervention compared to children in the control group. Children in the intervention group exhibited better results in writing, reading, and mathematics than their peers in the control group [11]. The second investigation observed that only boys in the intervention group improved their academic performance after nine years of intervention, whereas no differences were observed in girls. More specifically, a larger proportion of boys in the intervention group (96%) qualified for upper secondary school compared to boys in the control group (83%) [12].

Despite the fact that the limited available literature generally indicates a positive relationship between motor competence and academic performance, results are inconsistent with more studies being cross-sectional [8,14,15,16,17,18] than longitudinal [9,10] or experimental [11,12]. Moreover, it is not clear whether gross or fine motor competence skills are more closely related to academic performance. Studies have evaluated the association between motor competence and academic performance using overall measures of motor competence [12,19], only using gross [4,7,13,17] or fine [14] motor competence skills and both fine and gross motor competence skills in the same investigation [8,9,16,18]. Importantly, results are not consistent [4,7,8,9,12,13,14,16,17,18,19]. Thus, it is necessary to evaluate the association of fine and gross motor competence skills with academic performance.

Few investigations have evaluated factors that might mediate the association between motor competence and academic performance [17,18]. Kantomaa et al. (2013) observed that self-reported body mass index (BMI) and physical activity mediated the relationship between overall motor competence and academic performance in an eight-year longitudinal study. Although it was the first study to evaluate the possible mediation role of weight status, assessed by BMI, in the motor competence-academic performance relationship, the measures were self-reported, which is an important limitation of the study [17]. A similar study using objective measures would provide further evidence of associations. Furthermore, previous longitudinal investigations have shown waist circumference to be related to academic performance in children and adolescents and to mediate part of the relationship of physical activity and fitness with academic performance [20,21]. It is possible that central adiposity, represented by waist circumference, would be more closely related to motor competence (or at least gross motor skills) [22].

Thus, the purpose of the study was to evaluate the longitudinal association between motor competence (as assessed by the dynamic balance and a precision throwing task) and academic performance. A second purpose was to examine whether waist circumference, as a measure of obesity mediated this relationship. We hypothesize that gross and fine motor competence will be associated with academic performance and that waist circumference will mediate part of the relationships.

## 2. Materials and Methods

### 2.1. Design and Participants

The current study was part of the Childhood Health, Activity, and Motor Performance School Study Denmark (CHAMPS-study DK), a quasi-experimental study started in 2008. The present study was part of the Childhood Health, Activity and Motor Performance School Study Denmark (CHAMPS DK) study. All 19 government-funded schools in the municipality of Svendborg, Denmark, were invited to participate in the project by the municipality. Six of the schools agreed to be intervention schools, and the municipality and researchers decided that this was an adequate number for the purposes of the study. We had hoped to recruit six matching control schools, but only four schools agreed to take part. The complete methodology of the CHAMPS-study DK has been published extensively elsewhere [23]. Therefore, only variables of interest are described here. The study was conducted in accordance with the Declaration of Helsinki, approved by the local scientific ethics committee (ID S20080047 and S-20140105) and registered in the Danish Data Protection Agency (J.nr. 2008-41-2240). Parents had to provide written consent before their children were included in the study, and participants could decline their participation verbally.

### 2.2. Measurements

For this study, two data collections (spring 2010 and spring 2011) were used for the analysis. Participants were 1020 children from first (mean age 7.87 ± 0.34 years) through fifth grade (mean age 11.87 ± 0.37 years). Results from the Danish national academic performance tests performed in 2010 and 2011 were used as the outcome measure. The academic performance tests were standardized and performed on a computer. The test questions progressed according to the students’ performance and advanced to more complex questions after a correct answer, with less complex questions provided after an incorrect answer. For this study, tests in Danish language in second and fourth grades and math performance in third grade were used for the analyses. Each academic subject was assessed in three different domains (the score for each domain could range from zero to 100 points and the total test score from zero to 300). In particular, language understanding, decoding and text comprehension were domains evaluated in the Danish tests, and algebra, geometry and basic mathematics skills were the domains tested in Math. In sixth grade, an average of the performance in Danish and math was used because participants were tested in both subjects. In the current study, academic performance is the composite score of all domains in both Danish and math.

Mother’s education was collected via a questionnaire and categorized into five levels: primary and lower secondary education, general upper secondary education, vocational education and training, bachelor’s degree, and masters or PhD degree. Data were collected on the variables of interest for this study in spring of 2010 and 2011. Before all the test rounds, experienced researchers conducted training workshops for all testers, who comprised Bachelor, Masters, and PhD students from the University of Southern Denmark. The researchers tested their skills on fellow testers, with around 15 people in each test round, and performed the tests on children in similar age groups in schools outside of the Municipality of Svendborg. In addition, a detailed manual was provided for all measurements. Height was measured to the nearest 0.5 cm (cm) with a portable stadiometer (Seca 214, Seca Corporation, Hanover, MD, USA). Body mass was measured to the nearest 0.1 kg on an electronic scale (Tanita BWB-800S; Tanita Corporation, Tokyo, Japan) with the participant wearing light clothes. Waist circumference (WC) was measured with a measuring tape at the umbilicus level to the nearest 0.5 cm. Two WC measurements were performed, and in cases where the difference between the first and the second measurement was greater than 1.0 cm, a third measurement was performed. The average of the two nearest measurements was calculated and used for the analysis. 

Dynamic balance was assessed using a test from the “Körperkoordinationstest für Kinder” (KTK) [24]. Participants walked backward on balance beams of decreasing width (6.0, 4.5, and 3.0 cm), and trials were scored as the number of successful footsteps with a maximum of eight points per trial. The score on the dynamic balance test could range from 0–72 points. A precision throw test was used to assess an object projection skill (eye-hand-coordination—perceptuomotor integration), where each participant had two sets of five throws to hit a target plate standing three meters away. The precision throw score could range from 0–3 points per throw depending where the ball hit the plate (closer to the center: higher score), and 30 points was the highest score possible after all trials [25].

### 2.3. Data Analysis

For descriptive purposes, means and standard deviations are presented, and analysis of variance (ANOVA) was performed to test for differences in the descriptive characteristics between boys and girls and among grades (Table 1). For all analysis, we accepted 5% type I error. Structural equation modeling (SEM) with maximum likelihood for missing values (mlmv method in Stata) to not exclude participants because of a missing information was used to determine whether (i) balance and precision throw were longitudinally associated with academic performance and (ii) WC mediated the association between balance and precision throw with academic performance. STATA version 14.0 (StataCorp LP, College Station, TX, USA) was used for all analyses. All the association coefficients presented were standardized, and all the analyses were stratified by sex. Since preliminary analysis did not show relevant differences in the associations between the exposures (balance and precision throw) and math and language scores, we are analyzing math and language scores combined representing academic performance. 

**Figure 1:**
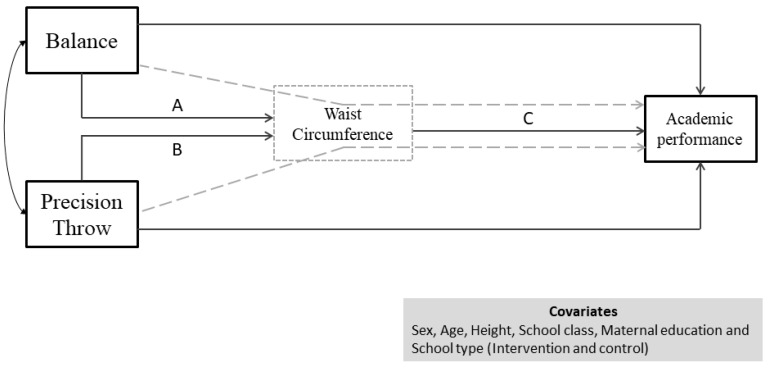
Simplified diagram of the pathways used in the analyses. WC refers to waist circumference. School type refers to assignment to control or intervention school physical education program. Academic performance refers to the grades in Danish and math combined. A is the direct association coefficient between balance and waist circumference. B is the direct association coefficient between precision throw and waist circumference. C is the direct association coefficient between waist circumference and academic performance. Dotted gray lines represent the indirect (mediated) associations. The curved arrows represent covariates.

A simplified framework for the analysis is presented in Figure 1. Specifically, the analysis examined the longitudinal association between two aspects of motor competence (dynamic balance and precision throw) and academic performance. The same diagram also portrays the potential mediating role of WC on the association between motor competence (dynamic balance and precision throw) and academic performance. The indirect (mediated) association, represented by the dotted lines in Figure 1, was estimated by multiplying the coefficients of the direct associations between exposure and mediator, and mediator and outcome. For example, the association between balance and academic performance mediated via WC was calculated by multiplying the association coefficients A with C (Figure 1). Similarly, the association between precision throw and academic performance mediated via WC was calculated by multiplying the association coefficients B with C (Figure 1). The total association was the sum of the direct and mediated associations between motor competence tests and academic performance. Note that analyses were adjusted for sex, age, height, school class, maternal education and school type (intervention and control). We considered that our model fitted the data by the following criteria: Comparative Fit index (CFI) > 0.900 and Root Mean Square Error of Approximation (RMSEA) < 0.080.

## 3. Results

At the first measurement, older children exhibited higher waist circumference, body mass, and height as compared to younger children for both sexes and the total sample. In addition, balance and precision throw scores were higher for older children. Girls scored higher in the balance test and in the Danish test compared to boys (see Table 1).

**Figure 2:**
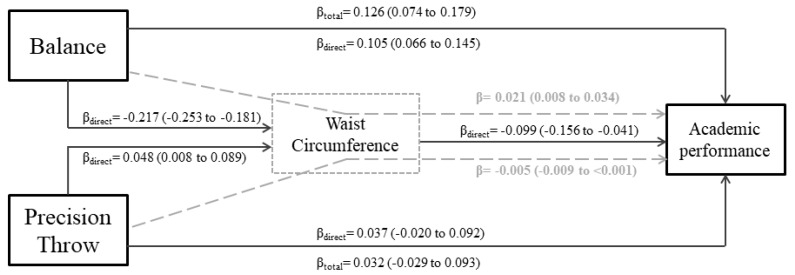
Standardized parameter estimates of the slopes (95% CI) of the direct, mediated, and total association between balance, precision throw and academic performance for all participants. Legend: Note that grey coefficients refer to indirect associations via waist circumference. βtotal refers to total association (direct and mediated association coefficients combined).

According to the post-estimation coefficients, the data demonstrated an adequate model fit (CFI = 0.969; RMSEA = 0.059). Our model showed a coefficient of determination of 0.759. In the total sample, balance was longitudinally associated with academic performance. Specifically, each additional standard deviation (SD) improvement in balance augmented the academic performance by 0.126 SD (total association), in which 0.105 is direct association from balance to academic performance and 0.021 SD improvement was mediated by waist circumference. Precision throw did not present total or mediated association with academic performance (see Figure 2).

In boys, dynamic balance was associated with academic performance (β_direct_ = 0.101; 95% CI: 0.051 to 0.152; β_total_ = 0.113; 95% CI: 0.050 to 0.176), but waist circumference did not mediate the association. For girls, balance presented direct (β = 0.098; 95% CI: 0.053 to 0.144), mediated (β = 0.025; 95% CI: 0.008 to 0.042—via waist circumference) and total association with academic performance (β = 0.123; 95% CI: 0.061 to 0.186). Thus, waist circumference mediated 20% of the total association between balance and academic performance. The strength of the total association between balance and academic performance was similar for boys (β = 0.113) and girls (β = 0.123). Precision throw was not associated with academic performance for boys or girls.

## 4. Discussion

We evaluated the longitudinal association between two aspects of motor competence and academic performance in Danish children. Balance was positively associated with academic performance, and the association was partially mediated by waist circumference. Furthermore, balance was associated with academic performance for both boys and girls individually. Alternatively, precision throw was not associated with academic performance.

It is possible that the synergistic associations among motor competence, physical activity, physical fitness and body weight [26] influence the tendency of whole body motor competence tasks to be more consistently related to academic performance than other, more specific motor competence tasks. Haapala et al. (2014) [9] observed the five meter shuttle run test to be longitudinally associated with academic performance, whereas aerobic capacity, assessed by a cycle ergometer test, was not related to academic performance. Although the five-meter shuttle run is also associated with aerobic fitness, the five-meter shuttle run test mainly assesses agility and speed. Thus, Haapala et al. (2014) hypothesized that the motor competence component in the shuttle run test might explain the selective association [9]. Lima et al. (2018) reported similar findings reinforcing the hypothesis that whole body motor competence tasks are more consistently related to academic performance than fine motor competence [21].

In the current study, motor competence, as assessed by dynamic balance, was associated with academic performance for both boys and girls. Although the strength of the total association for both sexes was similar, waist circumference partially mediated the relationship only for the girls. Data from this study and one additional study suggest that boys’ motor competence development may positively influence academic performance more than girls [11]. It is possible that the motor competence-academic performance relationship in girls may be more related to weight status, since 20% of the association between motor competence and academic performance in girls was mediated by waist circumference, whereas waist circumference did not mediate the association between balance and academic performance in boys.

We observed that waist circumference mediated the association between balance and academic performance. Kantomaa et al. (2013) [17] observed similar results indicating BMI mediated the association between motor competence, measured by a questionnaire, and academic performance during childhood and adolescence [17]. Kantomaa et al. (2013) [17] also reported physical activity level mediated the association between motor competence and academic performance; however, aerobic fitness was not found to mediate the motor competence-academic performance association [17]. Another study observed that working memory partially mediated the relationship between motor competence and academic performance [18]. The synergic relationship among motor competence, physical activity and fitness and body fatness [26] might explain the indirect relationship between balance and academic performance via waist circumference observed in the current study.

Higher motor competence leads to higher levels of physical activity and fitness and lower levels of body fat [26]. The positive relationship of physical activity and fitness with cognitive function and brain structure is relatively well-stablished [27,28] via changes in the functional and structural composition of the brain [29,30]. Moreover, the negative association between body fatness and brain structure and function has been described previously [31]. It has been shown that adolescents with metabolic syndrome exhibited lower academic performance, cognitive function and overall intelligence besides smaller hippocampal volumes, increased brain cerebrospinal fluid, and reductions of microstructural integrity in major white matter tracts [32]. Although only a few studies evaluated the relationship between motor competence and brain structure and function [5,33,34], it is possible that the motor competence association with physical activity, fitness, fatness and cognition are in the motor competence-academic performance relationship pathway.

The longitudinal design with a relatively large sample size and the extensive assessment of academic performance with both Danish and Math are strengths of the current study. The inclusion of waist circumference as a potential mediator also adds new information to the existing knowledge base. However, there are a number of limitations in the current study. This study is part of the CHAMPS-study DK, which was an intervention that increased the number of minutes per week the students were exposed to physical education classes in the intervention schools; however, the statistical analysis employed accounted for the intervention component. The participants were not tested for Danish and math every year; thus, the academic performance data could have been more extensive. Although the current study evaluated the association between motor competence and academic performance in a considerable age span (7–11 years of age at baseline), the follow-up of only one year was relatively short. Furthermore, only dynamic balance and precision throw were assessed as measures of motor competence; however, both assessments are representative of critical aspects of motor competence. Overall, examining only two specific motor competence tests limits the generalizability of the results.

## 5. Conclusions

Dynamic balance is an important gross motor function that was longitudinally related to academic performance, and waist circumference partially mediated the relationship. Precision throw was not found to be associated with academic performance. Future studies examining the relationship between motor competence and academic performance should assess other possible mediators (i.e., physical activity, fitness, and cognitive functions) and use more comprehensive assessments of motor competence to improve the understanding of underlying pathways. Our results and the results of others point to the importance of promoting the development of motor competence for all children, as data generally indicate it is positively related to academic performance as well as other health-related outcomes. Moreover, our study also supports the importance of adiposity, represented by waist circumference, on the relationship between gross motor function and academic performance during childhood.

## Figures and Tables

**Table 1:** Mean and standard deviation (SD) of the age, waist circumference (WC), weight, height, balance, precision throw and academic achievement scores for the different grade levels in 2010 for all participants and for boys and girls separately.

All Participants	First Grade		Second Grade		Third Grade		Fourth Grade		Fifth Grade	
	(n = 189–217)		(n = 225–299)		(n = 237–313)		(n = 238–313)		(n = 225–264)	
	Mean	(SD)	Mean	(SD)	Mean	(SD)	Mean	(SD)	Mean	(SD)
Age (years)	7.87	(0.34)	8.89	(0.33)	9.86	(0.39)	10.88	(0.32)	11.87	(0.37)
WC (cm) *	57.56	(5.32)	61.03	(6.22)	63.67	(6.90)	65.27	(7.17)	68.55	(8.27)
Weight (kg) *	27.05	(4.23)	30.96	(5.46)	34.13	(6.27)	37.83	(6.85)	42.46	(8.82)
Height (cm) *	129.35	(5.55)	135.72	(5.95)	140.61	(6.79)	146.27	(6.42)	152.26	(7.33)
Balance (points) *	40.59	(12.47)	42.35	(12.25)	49.00	(11.91)	54.69	(10.61)	54.18	(10.55)
Precision throw (points) *	9.95	(4.50)	12.48	(4.11)	14.86	(4.13)	16.92	(3.63)	17.91	(3.72)
Danish (points)	-	-	162.46	(75.53)	-	-	161.78	(75.39)	-	-
Math (points)	-	-	-	-	166.68	(66.52)	-	-	-	-
Boys	First grade		Second grade		Third grade		Fourth grade		Fifth grade	
Age (years)	7.90	(0.36)	8.96	(0.33)	9.94	(0.41)	10.95	(0.32)	11.94	(0.37)
WC (cm) *	57.38	(5.68)	61.24	(5.40)	63.68	(6.53)	65.50	(7.14)	68.93	(8.48)
Weight (kg) *	27.32	(4.28)	31.21	(4.94)	34.55	(6.07)	37.99	(6.93)	42.81	(9.33)
Height (cm) *	130.58	(5.76)	136.40	(5.74)	141.88	(6.97)	146.94	(6.56)	153.21	(7.34)
Balance (points) ^¥^	38.69	(11.69)	42.50	(13.43)	47.54	(11.64)	52.68	(10.53)	52.65	(11.30)
Precision throw (points)	11.66	(4.39)	13.96	(3.91)	16.44	(3.69)	17.99	(3.38)	18.84	(3.33)
Danish (points) ^¥^	-	-	166.94	(75.88)	-	-	154.82	(76.70)	-	-
Math (points)	-	-	-	-	168.75	(71.69)	-	-	-	-
Girls	First grade		Second grade		Third grade		Fourth grade		Fifth grade	
Age (years)	7.84	(0.33)	8.84	(0.32)	9.78	(0.35)	10.82	(0.32)	11.81	(0.35)
WC (cm) *	57.71	(5.03)	60.84	(6.85)	63.67	(7.31)	65.10	(7.22)	68.20	(8.10)
Weight (kg) *	26.82	(4.19)	30.73	(5.88)	33.66	(6.48)	37.71	(6.81)	42.13	(8.35)
Height (cm) *	128.33	(5.17)	135.14	(6.09)	139.21	(6.32)	145.75	(6.28)	151.38	(7.25)
Balance (points)	42.17	(12.92)	47.78	(10.61)	50.59	(12.04)	56.26	(10.45)	55.57	(9.66)
Precision throw (points)	8.54	(4.10)	11.20	(3.84)	13.12	(3.89)	16.08	(3.62)	17.06	(3.86)
Danish (points)	-	-	170.02	(70.03)	-	-	175.97	(72.28)	-	-
Math (points)	-	-	-	-	164.94	(58.83)	-	-	-	-

* *p* < 0.05 differences among grades; ^¥^ < 0.05 differences between boys and girls.
